# Improving Cardiometabolic Health with Diet, Physical Activity, and Breaking Up Sitting: What about Sleep?

**DOI:** 10.3389/fphys.2017.00865

**Published:** 2017-11-08

**Authors:** Grace E. Vincent, Sarah M. Jay, Charli Sargent, Corneel Vandelanotte, Nicola D. Ridgers, Sally A. Ferguson

**Affiliations:** ^1^Health, Medical and Applied Sciences, Central Queensland University, Rockhampton, QLD, Australia; ^2^Institute of Physical Activity and Nutrition, School of Exercise and Nutrition Sciences, Deakin University, Geelong, VIC, Australia

**Keywords:** exercise, physical activity, sitting breaks, sedentary behavior, sleep restriction

## Abstract

Cardiometabolic disease poses a serious health and economic burden worldwide and its prevalence is predicted to increase. Prolonged sitting, lack of physical activity, poor diet, and short sleep duration are ubiquitous behaviors in modern society, and all are independent risk factors in the development of cardiometabolic disease. Existing evidence demonstrates that breaking up prolonged periods of sitting is beneficial for cardiometabolic health, however, studies have not controlled for prior sleep duration. This article examines how prolonged sitting and short sleep duration independently contribute to cardiometabolic risk, and how breaking up sitting and obtaining adequate sleep may reduce this risk. We suggest that as prolonged sitting and short sleep duration influence the same cardiometabolic parameters, there is potential for short sleep to attenuate the positive impact of breaking up prolonged sitting with physical activity. Likewise, breaking up prolonged sitting and obtaining adequate sleep together could improve predictors of cardiometabolic disease, i.e., the combined effect may be stronger than either alone. To explore these perspectives, we propose a research agenda to investigate the relationship between breaking up prolonged sitting with physical activity and short sleep duration. This will provide an evidence-base for informing the design of interventions to reduce the burden of cardiometabolic disease on communities worldwide.

## Introduction: reducing the burden of cardiometabolic disease

Cardiometabolic disease describes a spectrum of conditions beginning with insulin resistance, progressing to the metabolic syndrome, pre-diabetes, and finally to more severe conditions including cardiovascular disease (CVD) and type 2 diabetes (T2DM) (Guo et al., 2014). These conditions are grouped under the umbrella term “cardiometabolic disease” as they are related or share risk factors, such as overweight and obesity, dyslipidemia, and high blood pressure (Vasudevan and Ballantyne, 2005). Alarmingly, 30% (2.1 billion) of the global population are either overweight or obese (Ng et al., 2014) and CVD is the leading cause of death worldwide (World Health Organization, 2015). In 2013, 382 million people had diabetes, with this number expected to rise to 592 million by 2035 (Guariguata et al., 2014). Further, the estimated annual global health expenditure attributable to diabetes averages $855 billion (USD) (da Rocha Fernandes et al., 2016) rising to $1044 billion (USD) by 2030 (Bloom et al., 2012). Thus, it is a public health imperative to reduce cardiometabolic risk and cardiometabolic disease development.

To reduce the risk of developing cardiometabolic disease, research and public health interventions have traditionally focussed on increasing physical activity, improving diet quality, and reducing tobacco use and alcohol intake (Cannon, 2007). While there have been significant reductions in tobacco use and alcohol intake (Australian Bureau of Statistics, 2017a,b), arguably little progress has been made in physical activity levels (Chau et al., 2017) and unhealthy diet behaviors (Australian Institute of Health and Welfare, 2017); lifestyle factors which are notoriously difficult to change (Dulloo et al., 2017). Dietary habits influence multiple risk factors for cardiometabolic health (Mozaffarian, 2016), and dietary factors have been shown to be associated with a substantial proportion of deaths from heart disease, stroke, and T2DM (Micha et al., 2017). Further, there is compelling evidence that engaging in 30–60 min a day of moderate-vigorous intensity physical activity (MVPA) improves health and reduces the prevalence of chronic disease (Warburton et al., 2006; Haskell et al., 2007; Oja and Titze, 2011). However, there is a curvilinear relationship between physical activity and health, which suggests that adults who do not engage in MVPA should do some activity for health benefits (Brown et al., 2013).

Focusing exclusively on promoting MVPA and improving diet, limits the potential to optimize health benefits of other lifestyle behaviors, such as low physical activity levels (e.g., standing, slow walking), sleep, and prolonged sitting (Chaput et al., 2014). Despite evidence highlighting that prolonged sitting and short sleep duration are modifiable risk factors for cardiometabolic disease (Mullington et al., 2009; Owen et al., 2010), historically they have received significantly less attention compared to physical activity and diet. A focus on increasing physical activity levels, diet, sleep duration, and reducing and/or breaking up prolonged sitting in combination is important in reducing cardiometabolic risk.

## Sedentary behavior, breaking up prolonged sitting, and cardiometabolic risk

Sedentary behavior is any waking behavior characterized by an energy expenditure ≤1.5 metabolic equivalents (METs), while in a sitting, reclining or lying posture (Barnes et al., 2012) (i.e., watching TV, using a computer, sitting in cars etc.). Sedentary behavior should be distinguished from “physical inactivity,” defined as an insufficient physical activity level to meet present physical activity recommendations (Tremblay et al., 2017). The opportunities for sedentary behavior, in particular prolonged sitting, are abundant in modern society. Objective estimates indicate that adults spend at least one half and up to two-thirds of their waking hours (or 8–10 h a day) engaged in sedentary behaviors (Healy et al., 2008; Matthews et al., 2008; Colley et al., 2011). Due to societal trends that move toward urbanization, car use, mechanization and technological advancements (Dunstan et al., 2010) in everyday tasks that involve sitting (e.g., sitting at work, using a computer, TV viewing, time spent sitting in vehicles), the prevalence of sedentary behaviors has increased.

Cross-sectional and prospective observational studies indicate that sedentary behavior is a risk factor for T2DM, CVD, and increased all-cause mortality (Wilmot et al., 2012), independent of physical activity levels (e.g., MVPA such as brisk walking/jogging/sports) (Bankoski et al., 2011; Thorp et al., 2011; Koster et al., 2012; Wijndaele et al., 2014). More specifically, time spent sitting is strongly associated with elevated rates of T2DM, metabolic syndrome, and obesity (Hamilton et al., 2007). Of concern, even in individuals who are otherwise physically active, a dose-response relationship exists between prolonged sitting and mortality (Katzmarzyk et al., 2009). Thus, an individual who is physically active may still be exposed to substantial health risks if they also engage in large amounts of occupational or recreational sitting. However, there is some evidence that high levels of MVPA (i.e., 60–75 min per day) eliminate the increased risk of death associated with high sitting time (Ekelund et al., 2016). However, for the majority of the population, the evidence supports cardiometabolic health benefits associated with reducing sitting time (Proper et al., 2011; Bauman et al., 2013).

In an effort to reduce sitting time, research over the last 5 years has focused on breaking up prolonged sitting at regular intervals (e.g., every 20–30 min) with short bursts (e.g., 2–3 min) of standing or short bouts of light-intensity physical activity (Benatti and Ried-Larsen, 2015). Breaking up prolonged sitting is associated with a significantly improved metabolic profile (Chastin et al., 2015), reduced self-reported fatigue (Wennberg et al., 2016), and reduced all-cause mortality risk (Katzmarzyk, 2014). Additionally, regularly breaking up prolonged sitting with short (1 min 40 s) bouts of walking (light-intensity physical activity) (Peddie et al., 2013) or standing (Benatti et al., 2017) is more effective than a single continuous (30 min) bout of MVPA per day in lowering postprandial glucose and insulin concentrations in healthy, normal weight adults.

The aforementioned epidemiological and experimental studies exploring the impact of breaking up prolonged sitting have prompted calls to revise public health physical activity and sedentary behavior guidelines. Specifically, too much sitting should be considered as a stand-alone component of the health equation, in addition to continuing to promote habitual engagement in MVPA (Kushi et al., 2006). In response, the UK, Canadian, and Australian physical activity guidelines have included statements on reducing sedentary behavior (Tremblay et al., 2011), with some explicitly recommending avoiding prolonged periods of sitting (Bull and Groups, 2010; Brown et al., 2013). In particular, the Australian guidelines specify to “break up long periods of sitting as often as possible.” The collective evidence highlights the importance of breaking up prolonged sitting to improve cardiometabolic health, however, the guidelines are based on studies that have not controlled for prior sleep duration, a key cardiometabolic disease risk factor.

## Sleep duration and cardiometabolic risk

Sleep is a basic requirement for human health. It serves critical physiological and psychological functions, including neurobehavioral performance (Killgore, 2010), metabolism (Copinschi et al., 2014), appetite regulation (Knutson, 2007), immune function (Besedovsky et al., 2012), and hormone regulation (Steiger, 2003). To maintain optimal health and functioning, a typical adult should obtain at least 7 h of sleep per night (Watson et al., 2015). However, as many as 45% of adults do not meet this recommendation (Centers for Disease Control Prevention, 2011; Adams et al., 2016). Epidemiological and experimental studies provide strong evidence for a link between sleep loss and adverse metabolic traits, including key components of cardiometabolic disease (e.g., obesity, T2DM, dyslipidemia, hypertension) (Knutson, 2010; Schmid et al., 2015). Further, a meta-analysis showed that less than 7 h of sleep per night was associated with increased risk of death (RR 1.12, 95% CI [1.06–1.18]) (Cappuccio et al., 2010). Another study reported a 57% increased risk (OR 1.57, 95% CI [1.11–2.22]) in T2DM development, even when adjusting for physical activity (Gangwisch et al., 2007). Collectively, this evidence suggests that short sleep should be targeted for prevention of cardiometabolic disease. Importantly, a recent review highlighted that most studies investigating the metabolic consequences of sleep disruption have enforced minimal physical activity (Potter et al., 2016). Future studies are needed to understand how to optimize exercise protocols to mitigate metabolic dysfunction caused by sleep disruption.

Sleep extension (increasing sleep) appears to benefit many aspects of cardiometabolic health. In habitually short sleeping adults (6.3 ± 0.5 h), increased time in bed (+44 ± 34 min) during a sleep extension intervention was associated with improved glucose regulation and insulin sensitivity (Leproult et al., 2015), a finding which has been observed in sleep extension interventions as short as 3 nights (Killick et al., 2015). Further, extending bedtimes of young overweight adults who habitually curtail their sleep has been associated with less desire for high calorie foods (Tasali et al., 2014). The collective evidence on the benefits of sleep extension suggests that increasing sleep duration can benefit cardiometabolic health in habitual short sleepers.

## Implications: do we need to examine sitting, diet, sleep, and physical activity behaviors in combination?

The aforementioned evidence suggests that prolonged sitting and short sleep not only influence the same cardiometabolic parameters associated with cardiometabolic disease risk, but do so independently. Experimental studies exploring the acute impacts of breaking up sitting on measures of cardiometabolic dysfunction, however, have not considered (recorded or controlled for) prior sleep duration. This is of concern because short sleep is associated with negative changes to the same cardiometabolic markers that positively change with breaking up prolonged sitting. Indeed, a recent study observed no benefit of breaking up prolonged sitting on glucose metabolism under conditions of sleep restriction (Vincent et al., in press). Therefore, there is potential for short sleep duration to attenuate the positive impacts of breaking up prolonged sitting. Further, obtaining adequate sleep or improving habitual sleep may further reduce cardiometabolic risk in those that regularly break up prolonged sitting. If short sleep does indeed offset the positive influence of breaking up sitting, this could have significant implications for workplaces and public health policy. For example, there has been recent and considerable investment in interventions that target prolonged sitting (e.g., implementing standing or treadmill desks). Yet if short sleep attenuates these effects, then recommendations relating to sleep should also be included in these interventions. By the same logic, improving an individual's sleep hours in combination with breaking up periods of prolonged sitting could further reduce cardiometabolic risk, i.e., the combined effect of adequate sleep and breaking up prolonged sitting may be stronger than either alone (i.e., a synergistic effect).

Short sleep, poor sleep quality and later bedtimes are associated with increased snacking (Nedeltcheva et al., 2009; Heath et al., 2012) food intake and poor diet quality (Chaput, 2014). In addition, obtaining adequate sleep is positively linked with healthy diet behaviors (Grandner et al., 2010). Reciprocally, unhealthy diet behaviors are associated with shorter sleep duration and irregular sleeping patterns (Peuhkuri et al., 2012). Since diet is a crucial component of normal glucose metabolism (Spiegelman and Flier, 2001), it is surprising that few studies have investigated how diet may influence postprandial glucose responses to breaking up prolonged sitting. A recent study found no difference between a high and standard energy diet in postprandial glucose responses in adolescents (Fletcher et al., in press). Further research is needed to investigate the impact of breaking up prolonged sitting and diet on cardiometabolic disease risk. Regardless, given the importance of diet in overall health, diet is critical when designing experimental or intervention studies to reduce cardiometabolic risk factors.

Time spent in sleep, sedentary behavior, and physical activity are co-dependent (Chastin et al., 2015). Spending time in one behavior means that an individual cannot be engaging in another behavior (Chastin et al., 2015). For example, if a sedentary individual decides to incorporate 30 min of physical activity into their day by waking up earlier, then total sleep time is reduced. Further, a reciprocity exists between sleep and physical activity (Buman et al., 2013). For example, engaging in physical activity may have positive effects on sleep (Benloucif et al., 2004; Dzierzewski et al., 2014), and improved sleep may increase an individual's ability to engage in physical activity the following day (Lambiase et al., 2013; Dzierzewski et al., 2014). Therefore, when reducing sedentary time, it is important to consider which behavior replaces it (i.e., sleep, light physical activity, MVPA) to optimize overall health.

Since behavioral impacts on health are unlikely to occur in isolation, interventions to change one behavior may not be sufficient to benefit health (James et al., 2016). Determining the optimal composition of a 24-h period to promote health and prevent chronic disease represents a new direction of behavior change intervention research (Chastin et al., 2015; Tremblay et al., 2016), beyond conventional approaches that advocate improving any single behavior in isolation (e.g., increasing amount of MVPA). Determining relationships across the entire spectrum of behaviors, from sleep to sitting time to physical activity, will provide health professionals and the community with coherent evidence-based guidance to tailor behavioral interventions for chronic disease risk reduction and public health promotion. To achieve this, investigating how behaviors interact with each other when they are combined is critical (e.g., prolonged sitting when sleep restricted), especially when determining the optimal composition of behaviors for overall health and well-being.

## Proposing a new research agenda

There are several key areas of investigation that are critical to this new research area. These include identifying the interactions between breaking up sitting and sleep hours, determining the critical composition of breaking up prolonged sitting (e.g., type, intensity, frequency) and sleep (e.g., duration, quality, timing) required for cardiometabolic health benefits, and the feasibility of interventions targeting both prolonged sitting and short sleep duration.

A series of rigorously-controlled laboratory protocols are required to investigate how breaking up prolonged sitting and sleep duration interact, and how this, in turn, impacts cardiometabolic health outcomes. Such protocols would involve determining how different combinations of sitting (duration and interruptions) and sleep (restricted, non-restricted) acutely affect markers of cardiometabolic health. Daily and postprandial measures of glucose concentration and insulin sensitivity (e.g., oral glucose tolerance tests) are required to assess the impact of these behaviors on glucose metabolism. Such studies should also attempt to determine the underlying physiological mechanisms behind observable changes in glucose metabolism through ancillary measures (e.g., endocrine levels, changes in gene expression).

There is still considerable debate concerning the optimal or minimum type, intensity, duration, and frequency of physical activity necessary to replace sitting time and to benefit cardiometabolic health (Benatti and Ried-Larsen, 2015). For example, those who choose to engage in physical activity 1 or 2 days of the week (termed the “weekend warriors”) may be sufficiently active to reduce all-cause, cardiovascular disease and cancer mortality risk regardless of adherence to current physical activity guidelines (O'donovan et al., 2017). Further, minimum physical activity requirements may need to be adjusted for individuals who routinely obtain insufficient sleep. For example, breaking up sitting with higher intensity physical activity or for longer durations may be required in those that are sleep restricted to achieve comparable health outcomes. Future research should also ascertain whether different dimensions of sleep, such as sleep efficiency, sleep quality and sleep timing, contribute to changes in glucose metabolism. Previous research has demonstrated that endocrine responses may be modulated by sleep timing; that is, early awakening and wakefulness during the second half of the night reduces early morning testosterone and prolactin levels, compared to the early part of the night showing no effect (Schmid et al., 2012). Further, the composition of sleep (i.e., sleep architecture) appears to influence glucose metabolism. For example, research in adults has demonstrated that selective suppression of slow wave sleep (i.e., deep sleep, non-rapid eye movement sleep) reduce measures of insulin sensitivity in a dose-dependent manner (Tasali et al., 2008) and can reduce postprandial insulin sensitivity by up to 20% (Herzog et al., 2013), suggesting slow-wave sleep may be important for normal glucose tolerance.

Changing behavior requires an understanding of why individuals engage in unhealthy behaviors (e.g., forgo sleep, make poor food choices etc.). While these reasons are often multi-factorial (e.g., work, society, family), such investigations inform intervention development. Intervention studies targeting multiple health behaviors simultaneously, in this case breaking up sitting and improving sleep, should be conducted to determine acceptability, feasibility, and short- and long-term efficacy (James et al., 2016). Interventions could consist of a series of behavior change sessions providing online personalized advice (Vandelanotte et al., 2015), added with commercially available sleep and activity trackers (e.g., Fitbit) that provide an objective indication of progress and that can also be used as tools to support behavior change (Salmon and Ridgers, 2012) (e.g., bedtime alarms to remind individuals to sleep, or prompts to break up prolonged sitting). Furthermore, infrastructure such as desks that permit standing or walking (e.g., sit-stand or treadmill desks) could be implemented at work, combined with improved sleep hygiene at home (e.g., reducing bedroom light exposure). More broadly, environmental and system level interventions could be implemented, such as engineering work settings to promote breaking up sitting (e.g., increasing distances between work stations) or providing workers with work start time flexibility to promote adequate sleep. Cardiometabolic outcomes could be assessed pre- and post- implementation of the behavior change intervention to break up sitting and improve sleep. Additionally, research grade sleep and activity monitors (i.e., accelerometers) that do not provide participants with feedback (to avoid reactivity) could be worn by workers to objectively determine to what extent behavior has changed (Vandelanotte et al., 2015).

The proposed research agenda will provide strong foundational evidence for a continuing line of research examining the impact of multiple health behavior change interventions for chronic disease prevention. This may provide a basis for the development of interventions to promote a mix of behaviors (e.g., regular physical activity, breaking up sitting, a healthy diet, adequate sleep) to benefit health.

## Conclusion

Effective prevention strategies that target physical activity, diet, sleep and sedentary behavior in combination are needed to improve cardiometabolic health. This includes an investigation into the interactions between behaviors, e.g., sleep, diet, prolonged sitting, and physical activity, to maximize health benefits and reduce cardiometabolic risk. As a first step, the behavioral duo of breaking up prolonged sitting *and* adequate sleep presents scope for optimization and intervention in the context of lifestyle-related chronic disease prevention. The application of the proposed research agenda will be wide-reaching and inform interventions to address the current burden of cardiometabolic disease, and will provide public health guidance by focusing on behaviors in combination.

## Author contributions

All authors contributed in writing the manuscript. GV wrote the first draft of the manuscript. SF provided critical feedback and revised the first draft. SJ, CS, CV, and NR contributed perspectives on their specific areas of expertise.

### Conflict of interest statement

The authors declare that the research was conducted in the absence of any commercial or financial relationships that could be construed as a potential conflict of interest.
